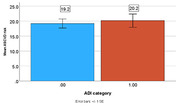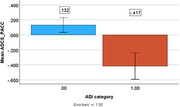# Neighborhood Disadvantage and Its Effects on Cognitive Health and Vascular Risk in Veterans

**DOI:** 10.1002/alz.094844

**Published:** 2025-01-09

**Authors:** Olivia Deering, Carol A. Van Hulle, Hannah Zylstra, Kate Cronin, Aleshia Cole, Elena Beckman, Allison C Eierman, Madeleine Blazel, Karen K Lazar, Kevin M. Johnson, Leonardo A. Rivera‐Rivera, Richard Chapell, Henrik Zetterberg, Carey E. Gleason, Sterling C. Johnson, Sanjay Asthana, Cynthia M. Carlsson

**Affiliations:** ^1^ Wisconsin Alzheimer’s Disease Research Center, University of Wisconsin‐Madison School of Medicine and Public Health, Madison, WI USA; ^2^ Wisconsin Alzheimer’s Disease Research Center, University of Wisconsin School of Medicine and Public Health, Madison, WI USA; ^3^ Alzheimer’s Disease Research Center, School of Medicine and Public Health, University of Wisconsin‐Madison, Madison, WI USA; ^4^ Geriatric Research, Education and Clinical Center (GRECC), William S. Middleton Memorial Veterans Hospital, Madison, WI USA; ^5^ Wisconsin Alzheimer’s Disease Research Center, University of Wisconsin School of Medicine and Public Health, Madison, WI, USA, Madison, WI USA; ^6^ School of Medicine and Public Health, University of Wisconsin‐Madison, Madison, WI USA; ^7^ University of Wisconsin‐Madison, School of Medicine and Public Health, Madison, WI USA; ^8^ Department of Biostatistics and Medical Informatics, University of Wisconsin School of Medicine and Public Health, Madison, WI USA; ^9^ Wisconsin Alzheimer's Disease Research Center, University of Wisconsin School of Medicine and Public Health, Madison, WI USA; ^10^ Alzheimer’s Disease Research Center, University of Wisconsin School of Medicine and Public Health, Madison, WI USA; ^11^ Alzheimer’s Disease Research Center, University of Wisconsin‐Madison School of Medicine and Public Health, Madison, WI USA

## Abstract

**Background:**

Prior research has highlighted the impact of neighborhood quality on health outcomes. Given veterans' unique experiences and challenges, exploring the association between neighborhood quality on cognitive measures and vascular risk scores is crucial for guiding targeted interventions, improving overall cognitive well‐being, promoting health equity, and contributing to our understanding of Alzheimer’s Disease (AD) risk factors.

**Method:**

The Brain Amyloid and Vascular Effects of Eicosapentaenoic Acid study (BRAVE) was an 18‐month randomized, placebo‐controlled, double‐blind, clinical trial conducted at William S. Middleton Memorial Veteran’s Hospital in Madison, Wisconsin. This analysis used cross‐sectional data on VA‐eligible Veterans between the ages of 50 and 75 with no clinical diagnosis of a memory disorder. Study analyses included *N* = 201 Veterans who completed pre‐study screening and *N = 129* who completed a baseline visit. Cognitive outcomes were collected at the baseline visit and ASCVD scores were collected during screening. Of the Veterans who completed a pre‐study screening, live in Wisconsin (N = 164), Illinois (N = 34), Iowa (N = 2) and Minnesota (N = 1). The main outcomes are ASCVD risk score and Preclinical Alzheimer’s Cognitive Composite (PACC). The Area Deprivation Index (ADI) state decile measures neighborhood quality on 18 metrics. For this study, participants in the most disadvantaged (ADI 7‐10) were compared to participants in the least disadvantaged neighborhoods (ADI 1‐6).

**Result:**

In the prescreening cohort, 87.7% of participants were male and 89.4% non‐Hispanic, white. In the baseline cohort, 84.5% of participants were male and 94.7% were non‐Hispanic, white. PACC scores were significantly lower (PACC; p = 0.007; Cohen’s D = 0.562) for participants living in the most disadvantaged neighborhoods (‐0.42, SD = 0.98, N = 31) compared to the least disadvantaged (0.13, SD = 0.97, N = 98). The group mean differences in ASCVD scores were not statistically significant.

**Conclusion:**

Our findings reveal that Veterans in the most disadvantaged neighborhoods exhibit lower cognitive performance. We found no significant association between neighborhood disadvantage and cardiovascular risk scores. These findings emphasize the importance of implementing targeted interventions to address cognitive health disparities in disadvantaged areas. Further exploration with a larger and more representative sample is essential to gain a comprehensive understanding of the relationship between neighborhood disadvantage, cognitive health disparities, and cardiovascular risk factors among Veterans.